# Thermal Annealing Effect on Structural, Morphological, and Sensor Performance of PANI-Ag-Fe Based Electrochemical *E. coli* Sensor for Environmental Monitoring

**DOI:** 10.1155/2015/696521

**Published:** 2015-05-20

**Authors:** Norshafadzila Mohammad Naim, H. Abdullah, Akrajas Ali Umar, Aidil Abdul Hamid, Sahbudin Shaari

**Affiliations:** ^1^Department of Electrical, Electronic and System Engineering, Faculty of Engineering and Built Environment, Universiti Kebangsaan Malaysia, 43600 Bangi, Selangor, Malaysia; ^2^School of Applied Physics, Faculty of Science and Technology, Universiti Kebangsaan Malaysia, 43600 Bangi, Selangor, Malaysia; ^3^School of Biosciences and Biotechnology, Faculty of Science and Technology, Universiti Kebangsaan Malaysia, 43600 Bangi, Selangor, Malaysia; ^4^Institute of Microengineering and Nanoelectronics, Universiti Kebangsaan Malaysia, 43600 Bangi, Selangor, Malaysia

## Abstract

PANI-Ag-Fe nanocomposite thin films based electrochemical *E. coli* sensor was developed with thermal annealing. PANI-Ag-Fe nanocomposite thin films were prepared by oxidative polymerization of aniline and the reduction process of Ag-Fe bimetallic compound with the presence of nitric acid and PVA. The films were deposited on glass substrate using spin-coating technique before they were annealed at 300°C. The films were characterized using XRD, UV-Vis spectroscopy, and FESEM to study the structural and morphological properties. The electrochemical sensor performance was conducted using *I-V* measurement electrochemical impedance spectroscopy (EIS). The sensitivity upon the presence of *E. coli* was measured in clean water and *E. coli* solution. From XRD analysis, the crystallite sizes were found to become larger for the samples after annealing. UV-Vis absorption bands for samples before and after annealing show maximum absorbance peaks at around 422 nm–424 nm and 426 nm–464 nm, respectively. FESEM images show the diameter size for nanospherical Ag-Fe alloy particles increases after annealing. The sensor performance of PANI-Ag-Fe nanocomposite thin films upon *E. coli* cells in liquid medium indicates the sensitivity increases after annealing.

## 1. Introduction

*Escherichia coli* (*E. coli*) bacteria can be found in contaminated water and food.* E. coli* are harmless and cause relatively brief diarrhea. But a few particularly nasty strains such as* E. coli* O157:H7 can cause severe abdominal cramps, bloody diarrhea, and vomiting.* E. coli* is being widely chosen as an indicator species of bacteria since it is fecal coliform bacteria that are specific to the intestines of human and other warm-blooded animals but not normally pathogenic, it is easy to detect and culture and it is found at higher concentrations than other pathogens in waters [1].

To detect* E. coli* in water, biosensors which offer simple, rapid, sensitive, and selective detection method for analysis of environmental contaminants are highly required. Three common types of biosensors have been developed for bacteria determination based on electrochemical [2–4], optical [5, 6], piezoelectric [7, 8] detection. Electrochemical biosensors are based on selective interaction between target analyte and recognition element, including potentiometric, voltammetric, amperometric, and electrochemical impedance spectroscopy biosensors. The interaction can produce an electrical signal that is related to the concentration of the analyte being studied [9].

Polyaniline (PANI) is found to be the most promising because of its easy synthesis, low-cost monomer, tunable properties, high conductivity, and better stability compared to others. The conductivity of PANI can be controlled by the process of doping which may be carried out through a chemical, electrochemical, or photochemical route [10]. Interest in metal nanoparticles was motivated by their potential applications of electrochemical sensor, due to their small size (1 nm–100 nm), unique chemical, physical, and electronic properties, and flexibility to construct novel and improved sensing devices [11]. Ag-Fe alloy is such a two-component alloy system consisting of magnetic iron (Fe) and nonmagnetic silver (Ag). Both Ag and Fe are safe for human cells, but lethal for bacteria and viruses. Reduction of the particles size of the materials is an efficient and reliable tool for improving their biocompatibility that can be achieved using nanotechnology [12]. The interaction between polyaniline and metals has attracted a great deal of interest in a wide variety of applications. When metal ions are added into the synthesized system of polyaniline, the metal ions can interact with the nitrogen atoms in the polyaniline chains [13] and hence increase the electrical properties of the system and become more suitable for fabrication of biosensor.

In this paper, the nanocomposite thin films of PANI and Ag-Fe alloy nanoparticle were synthesized by sol-gel method using spin-coating technique. Various compositions of Ag-Fe alloy nanoparticles were synthesized to study the optimum composition for the sensor to perform high sensitivity. The effects on structural, morphological, and sensor performance were studied before and after thermal annealing. A simple and low-cost prototype of PANI-Ag-Fe nanocomposite thin films based microbial sensor was fabricated and conducted using *I*-*V* measurement and electrochemical impedance spectroscopy (EIS) to detect* E. coli* in water.

## 2. Materials and Methods

### 2.1. Reagents and Materials

The precursor of silver nitrate (AgNO_3_, 99.99% purity), iron nitrate (Fe(NO_3_)_3_ nanohydrate ACS reagent >98%), aniline monomer (C_6_H_5_NH_2_), and polyvinyl alcohol (PVA, 99% hydrolysis) was purchased from Sigma-Aldrich Chemicals. A strain of* E. coli* O157:H7 was obtained from microbiological laboratory, Universiti Kebangsaan Malaysia.

### 2.2. Synthesis of Samples

2.5 g of PVA was completely dissolved in 40 mL deionized water and stirred on the hot plate at 80°C–90°C. The mixture of AgNO_3_ and Fe(NO_3_)_3_ was varied using the composition formula Ag_*x*_-Fe_1−*x*_ (*x* = 0.8, 0.6, 0.5, 0.4, and 0.2) and was dissolved in deionized water. The percentages of metals composition were listed in Table 1. Then, the Ag-Fe alloy was added drop by drop into PVA solution and it was continuously stirred until the colour of solution becomes golden brown. 1.25 mL of aniline was added to the solution followed by 1.0 M nitric acid (HNO_3_). The mixture was stirred until the solution changed to a greenish dark liquid indicating that the solution becomes PANI-Ag-Fe nanocomposite. The nanocomposite solution was spin-coated onto glass substrate using Laurell Technologies Corporation photoresist spinner, with the speed of 2000 rpm for 15 s. The films were annealed in a tube furnace at maximum temperature 300°C.

### 2.3. Fabrication of Sensors

**Figure 1:**
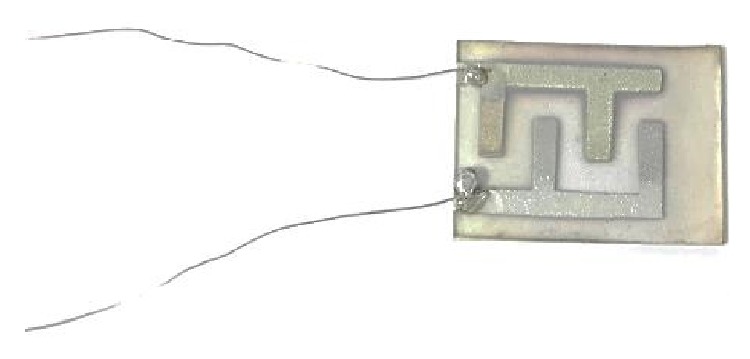
The fabricated PANI-Ag-Fe thin film sensor device.

The film size is 20 mm × 25 mm. The size of silver electrode has been measured to be 2 mm of width and the separation between two combs of electrode is 3 mm. As shown in Figure 1, a comb-structure of silver electrode was sputtered on the PANI-Ag-Fe films by using magnetron sputtering equipment. Argon gas was supplied in the sputtering chamber with the output power of 50 W within 454 s to sputter 1000 Å thickness of silver film. Cu wires were soldered to the silver electrodes as the connection between sensor electrode and the measuring device.

### 2.4. Characterization

X-ray diffraction (XRD) analysis was conducted on Bruker model D8 advanced X-ray diffractometer using CuK_*α*_ radiation (*λ* = 1.5406 Å) and the measurement was performed in 2*θ* range from 20° to 60°. The optical characterization of PANI-Ag-Fe thin films was carried out using Perkin Elmer Lambda 950 UV-visible spectroscopy in 300 nm to 800 nm wavelength. Surface morphology of the films was studied from Atomic Force Microscopy (AFM). Structural morphology was conducted using Field Emission Scanning Electron Microscopy (FESEM).

### 2.5. Impedance Measurement and Sensitivity Performance

Electrochemical impedance spectroscopy (EIS) of the sensor electrode was carried out with an applied potential of 1 V across the electrodes over the frequency 50 kHz–1 Hz with GAMRY-Physical Electrochemistry instrument. The sensing process was carried out by immersing the sensor electrode into the sample of clean water and water containing 10^8^ CFU mL^−1^ concentration of* E. coli* O157:H7.

## 3. Results and Discussion

**Figure 2:**
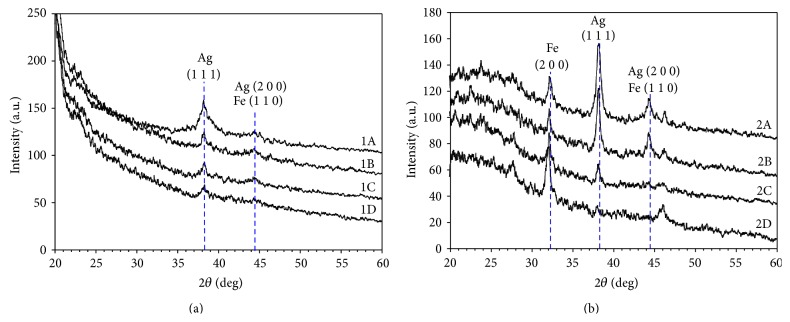
XRD patterns of PANI-Ag-Fe nanocomposite thin films samples (a) before annealing and (b) after annealing.

The X-ray diffraction (XRD) patterns of samples 1A–1D and 2A–2D were shown in Figure 2.  Figure 2(a) shows abroad peak of Ag and Fe while Figure 2(b) shows narrow peaks which indicate the high degree of crystallinity of Ag and Fe. Bragg's reflections at 2*θ* = 32.1°, 38.3°, and 44.5° correspond to the face centered cubic structure of Fe $\left(\begin{array}{ccc} 2 & 0 & 0 \end{array}\right)$, Ag $\left(\begin{array}{ccc} 1 & 1 & 1 \end{array}\right)$, and Ag-Fe $\left(\begin{array}{ccc} 1 & 1 & 0 \end{array}\right)$ embedded in PANI matrix. The indexes were obtained from JCPDS file with PDF numbers 01-087-0717 and 00-001-1262. In Figure 2(b), the peak of Fe $\left(\begin{array}{ccc} 2 & 0 & 0 \end{array}\right)$ appears whereas the peak of Fe $\left(\begin{array}{ccc} 1 & 1 & 0 \end{array}\right)$ appears more clearly which means that these Fe crystals formed at high temperature. The intensity of Ag and Fe peaks is sharper and higher when their concentration percentage in the sample increased. The crystallite size, *D*, was being calculated using Scherrer equation [14]:

(1) $$\begin{array}{c} D=\frac{0.9\lambda }{\beta \cos\theta }, \end{array}$$

where *D* is crystallite size, *λ* is X-ray wavelength (1.5406 Å), *β* is full width at half maximum (FHWM), and *θ* is the diffraction angle. The values of the calculated crystallite size have been summarized in Table 2. The crystallite sizes increase directly with Fe concentration. The crystallite sizes of the sample also increase after annealing at 300°C. It shows that high temperature exposed to the materials can enhance the growth of particles inside it.

**Figure 3:**
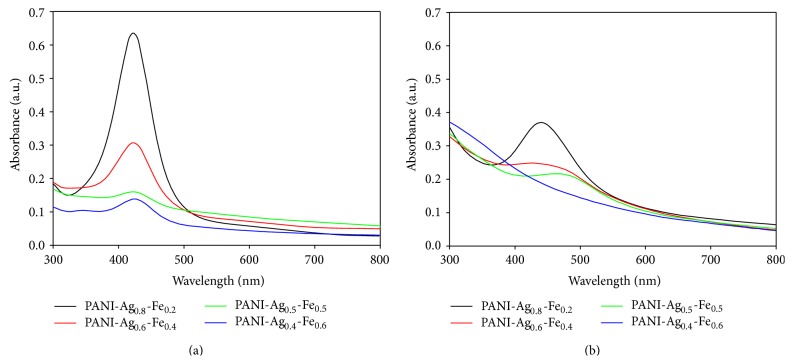
UV-Vis absorption bands of PANI-Ag-Fe nanocomposite thin films (a) before annealing and (b) after annealing.

Figure 3 shows the absorption bands of PANI-Ag-Fe nanocomposite thin films from UV-Vis spectroscopy analysis in various types of Ag-Fe alloy composition. The appearance of single absorbance peaks at 422–424 nm for Figure 3(a) and 426–464 nm for Figure 3(b) indicates that present Ag-Fe bimetallic particles are in alloy form rather than being a mixture of individual metal particles. From the figure, it can be seen that the absorbance intensity is higher for Ag-rich Ag-Fe alloy and lower for Fe-rich Ag-Fe alloy. The increasing of absorbance intensity indicates the formation of more nanoparticles [15]. So the higher absorbance intensity for PANI-Ag_0.8_-Fe_0.2_ in both figures reflects the formation of more Ag-Fe alloy nanoparticles in the sample. In comparison of samples before and after annealing, the absorbance peaks in Figure 3(b) shift to the longer wavelength than Figure 3(a) which indicates that the size of particles becomes larger. This is because the larger particles require lesser energy and hence longer wavelength [15]. So the samples after annealing at 300°C produce larger particle size.

**Figure 4:**
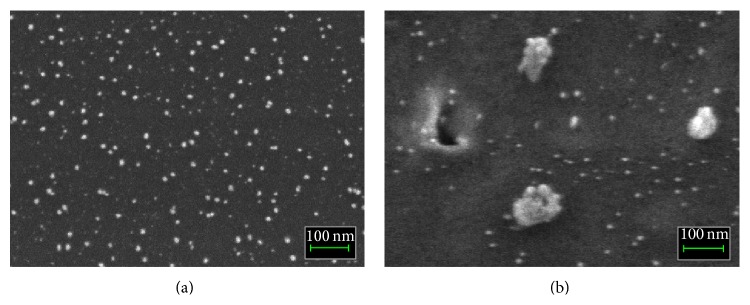
FESEM image of PANI-Ag_0.2_-Fe_0.8_ nanocomposite thin film (a) before annealing and (b) after annealing.

Figure 4 shows the internal structure images of PANI-Ag_0.2_-Fe_0.8_ nanocomposite thin films from FESEM analysis. Both images indicate the existence of nanospherical Ag-Fe alloy particles in the PANI matrix.  Figure 4(a) is the image of PANI-Ag_0.2_-Fe_0.8_ nanocomposite thin films before annealing where the samples were not exposed to the high temperature. In the figure, the nanoparticles of Ag-Fe alloy are well-dispersed and the size is smaller with average diameter of about ~5 nm to ~25 nm.  Figure 4(b) is the image of PANI-Ag_0.2_-Fe_0.8_ nanocomposite thin films after annealing. The size of nanoparticles can be seen larger than that in Figure 4(a) which is around ~10 nm to ~40 nm in diameter and some of the particles are agglomerated. This result also verified the result from UV-Vis and the trend of the particle size is corresponding to the result of crystallite size from XRD analysis.

**Figure 5:**
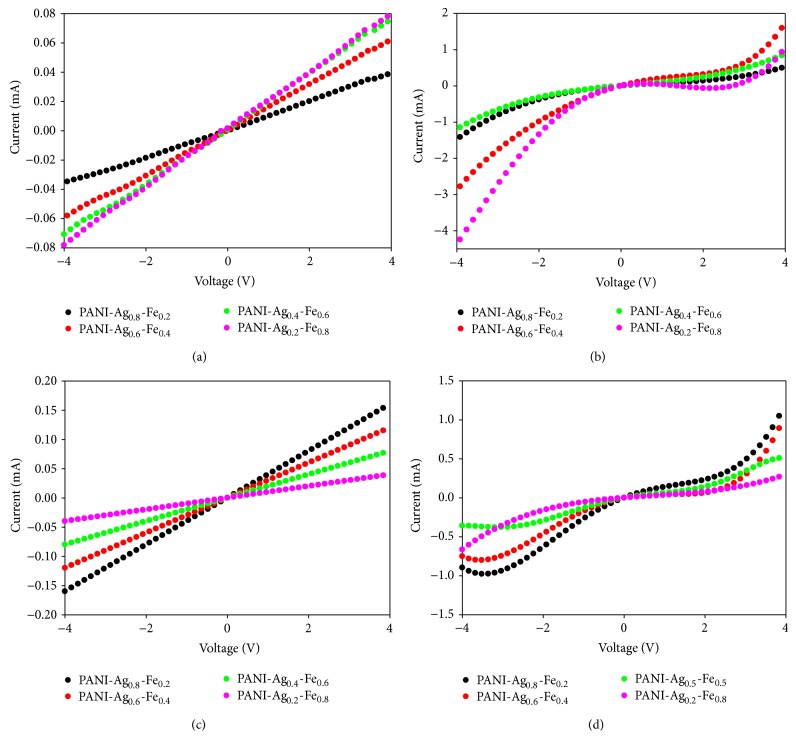
*I*-*V* measurement for PANI-Ag-Fe thin film sensor for the samples before annealing, (a) without* E. coli*, (b) with* E. coli*, and for the samples after annealing, (c) without* E. coli*, (d) with* E. coli*.

The performance of the prototype biosensor has been measured through the *I*-*V* measurement to study the current change of the thin film sensor with and without incubation to* E. coli*.  Figure 5 shows the change of current for each of PANI-Ag-Fe nanocomposite thin film samples in different conditions which are in clean water and water with* E. coli*. The current is apparently changed when the sensor electrode was immersed from clean water to* E. coli* solution. This proves the existence of reactions between metal and microbe. The metal and microbe interactions are mainly related to the cell wall and outer membrane arrangement. This is due to the significant differences in the outer layers of gram-negative and gram-positive bacteria. The cell wall of gram-negative bacteria consists of lipids, protein, and lipopolysaccharides (LPS) that ensure more effective defense against biocides in comparison to gram-positive bacteria where the cell wall does not contain outer membrane of LPS [16]. Since* E. coli* are gram-negative bacteria, they possess an outer membrane and a unique periplasmic space [17]; thus* E. coli* are more susceptible to Ag-Fe alloy nanoparticles.

**Figure 6:**
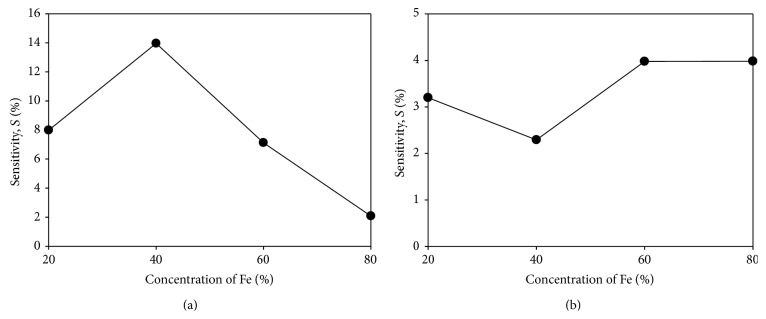
Sensitivity (*S*) upon* E. coli* for PANI-Ag-Fe nanocomposite thin films against concentration percentage of Fe (a) before annealing and (b) after annealing.

The sensitivity (*S*) of a sensor is described as the ratio of the magnitude of response upon exposure to the microbe (*I*_*e*_) to that without exposure to the microbe (*I*_*o*_).  Figure 6 shows the graph of sensitivity (*S*) on* E. coli* against the annealing temperature of PANI-Ag-Fe nanocomposite thin films which is calculated using the following formula [18]:

(2) $$\begin{array}{c} S=\frac{\left(I_{e}-I_{o}\right)}{I_{o}}\times 100, \end{array}$$

where *S* is the sensitivity of sensor electrode on* E. coli*, *I*_*e*_ is the current when the sensor electrode is exposed to* E. coli,* and *I*_*o*_ is the current when the sensor electrode is not exposed to* E. coli*.  Figure 6(a) shows that the maximum sensitivity is performed by PANI-Ag_0.6_-Fe_0.4_, whereas, in Figure 6(b), the maximum sensitivity is performed by PANI-Ag_0.2_-Fe_0.8_. From Figure 6, it also can be observed that the sample before annealing produces higher sensitivity compared to the sample after annealing. This is due to the characteristics of the thin films after annealing which produce larger size of crystallites and particles which is not compatible with a biosensor.

**Figure 7:**
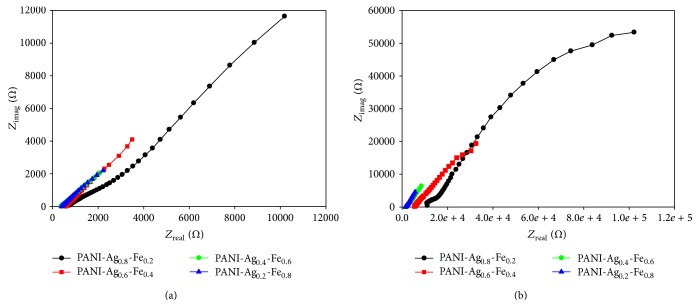
Nyquist impedance plot of PANI-Ag-Fe nanocomposite thin films microbial sensor (a) before annealing and (b) after annealing, when incubated in* E. coli* solution.

**Figure 8:**
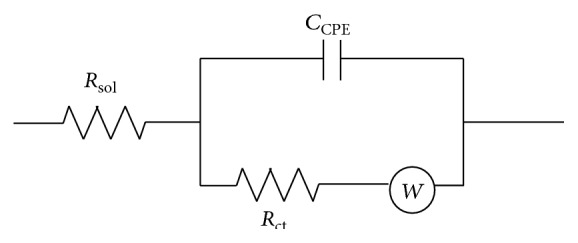
Equivalent electrical circuit.

The Nyquist impedance plots of the as-deposited PANI-Ag-Fe nanocomposite thin films with annealing temperature of 300°C when the films were immersed into* E. coli* bacteria solution are shown in Figure 7. *Z*_real_ is the real part and *Z*_imag_ is the imaginary part of the complex impedance over the frequency range 1 Hz–50 kHz with AC amplitude of 1 V. The equivalent RC model for the polymer-metal film is shown in Figure 8 where *R*_sol_ is the solution resistance, *C*_CPE_ is a constant phase element capacitance, *W* is the Warburg impedance, and *R*_ct_ is the charge transfer resistance at the polymer-metal film interface. The equivalent circuit was considered in order to analyze the impedance spectroscopy data. In the circuit, the element *W* is related to the ion transfer phenomenon [19]:

(3) $$\begin{array}{c} Z_{W}=\frac{\sigma \left(1-j\right)}{\omega^{1/2}}. \end{array}$$

The constant phase element (CPE) is used for the diffusion at low frequencies and it is defined as [19]

(4) $$\begin{array}{c} Z_{\text{CPE}}=\frac{1}{\text{CPE}{\left(j\omega \right)}^{\alpha }}. \end{array}$$

Figure 7(a) is related to PANI-Ag-Fe nanocomposite thin film before annealing at 300°C shows the impedance data in the range 64 Ω–2 kΩ for all concentration of Ag-Fe alloy. The impedance data range increases to 8 Ω–53 kΩ as shown in Figure 7(b) for PANI-Ag-Fe after annealing at 300°C. These impedance differences in the cell system were obtained by changing the thin film samples in the sensor system where the films were immersed into water with* E. coli*.

Total impedances were obtained from the total of real impedance and imaginary impedance using this equation:

(5) $$\begin{array}{c} Z_{\text{total}}=\sqrt{Z_{\text{real}}^{2}+Z_{\text{imag}}^{2}}. \end{array}$$

**Figure 9:**
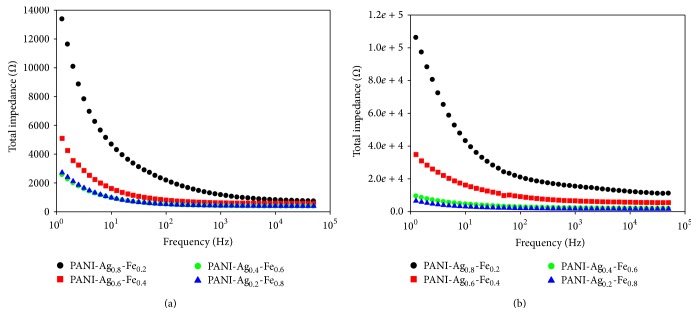
Total impedance versus frequency plots when incubated in* E. coli* solution for PANI-Ag-Fe nanocomposite thin films (a) before annealing and (b) after annealing at 300°C.

PANI-Ag-Fe nanocomposite thin films before and after annealing were exposed to* E. coli* in water and the impedance values have been measured. The increase in the impedance with increasing frequency can be attributed to capacitive component of the current [20]. The impedance value at high frequency increases after bacteria exposure which indicates that the increase in *R*_ct_ dominates the total impedance value in the high frequency range. From Figure 9, the higher the Fe concentration was, the lower the impedance was measured. In Figure 9(a), PANI-Ag-Fe thin film before annealing performed the lowest impedance due to the smaller size and better dispersion of the grain inside the nanocomposite. The impedance increases with the thermal annealed sample using vacancy hopping. As the annealing temperature increases, the dissolution of Ag might be associated with an intensive diffusion current of vacancies. This causes the electrical impedance change to increase with increasing of the concentration of vacancies which rises gradually with increasing annealing temperature [21].

## 4. Summary

PANI-Ag-Fe nanocomposite thin films based electrochemical* E. coli* sensor was fabricated for environmental monitoring application. Various compositions of Ag-Fe alloy nanoparticles were synthesized to study the optimum composition for the sensor to perform high sensitivity. The effects on structural, morphological, and sensor performance were studied before and after thermal annealing. XRD analysis shows that the crystallite sizes were found to become larger for the samples after annealing at 300°C. UV-Vis absorption bands for samples before and after annealing show maximum absorbance peaks at around 422 nm–424 nm and 426 nm–464 nm, respectively, and it also proves that the samples after annealing produce larger particles. FESEM shows the changing in diameter size for nanospherical Ag-Fe alloy particles from 5 nm–25 nm before annealing to 10 nm–40 nm after annealing. The sensor performance of PANI-Ag-Fe nanocomposite thin films upon* E. coli* cells in liquid medium indicates the sensitivity increases after annealing at 300°C.

## Figures and Tables

**Table 1:** Composition of metals in PANI-Ag-Fe nanocomposite.

Sample	Molar ratio of AgNO_3_ : Fe(NO_3_)_3_ in PANI-Ag-Fe	Ag content (molar%)	Fe content (molar%)	Annealing temperature (°C)
1A	PANI-Ag_0.8_-Fe_0.2_	80	20	—
1B	PANI-Ag_0.6_-Fe_0.4_	60	40	—
1C	PANI-Ag_0.4_-Fe_0.6_	40	60	—
1D	PANI-Ag_0.2_-Fe_0.8_	20	80	—
2A	PANI-Ag_0.8_-Fe_0.2_	80	20	300
2B	PANI-Ag_0.6_-Fe_0.4_	60	40	300
2C	PANI-Ag_0.4_-Fe_0.6_	40	60	300
2D	PANI-Ag_0.2_-Fe_0.8_	20	80	300

**Table 2:** Crystallite sizes for PANI-Ag-Fe before and after annealing.

Sample	Crystallite size before annealing (nm)	Crystallite size after annealing (nm)
PANI-Ag_0.8_-Fe_0.2_	18.17	26.90
PANI-Ag_0.6_-Fe_0.4_	21.69	33.62
PANI-Ag_0.4_-Fe_0.6_	29.24	35.39
PANI-Ag_0.2_-Fe_0.8_	39.56	42.00
